# Physicians’ perceptions of medical representative visits in Yemen: a qualitative study

**DOI:** 10.1186/1472-6963-13-331

**Published:** 2013-08-20

**Authors:** Mahmoud Abdullah Al-Areefi, Mohamed Azmi Hassali, Mohamed Izham b Mohamed Ibrahim

**Affiliations:** 1 Ministry of Public Health and Population, Health Management Training Center, Sana’a, Republic of Yemen; 2 School of Pharmaceutical Sciences, Universiti Sains Malaysia, Penang, Malaysia; 3 Social & Administrative Pharmacy, College of Pharmacy, Qatar University, Doha, Qatar

**Keywords:** Medical representatives, Information, Yemen, Qualitative study

## Abstract

**Background:**

The pharmaceutical industry invests heavily in promotion, and it uses a variety of promotional strategies to influence physicians’ prescribing decisions. Within this context, medical representatives (MRs) are the key personnel employed in promoting their products. One significant consequence of the interactions between physicians and medical representatives is a conflict of interests which may contribute to the over prescribing of medications and thus negative effects on patients’ health and economics. There is limited detailed information published on the reasons why physicians interact with pharmaceutical representatives. This study aims to qualitatively explore physicians’ attitudes about interactions with medical representatives and their reasons for accepting the medical representatives’ visits.

**Methods:**

In-depth interviews were used to gain a better understanding of physicians’ perceptions of medical representative visits. A total of 32 physicians from both private and public hospitals were interviewed. The recordings of the interviews were transcribed verbatim and subject to thematic analysis using a framework analysis approach.

**Results:**

The present qualitative study found that the majority of the physicians had positive interactions with medical representatives. The physicians’ main reasons stated for allowing medical representatives’ visits are the social contacts and mutual benefits they will gain from these representatives. They also emphasized that the meeting with representatives provides educational and scientific benefits. A few physicians stated that the main reasons behind refusing the meeting with medical representatives were lack of conviction about the product and obligation to prescribe medicine from the representative company. Most of the physicians believed that they were under marketing pressure to prescribe certain medicines.

**Conclusions:**

Although physicians are aware that the medical representatives could influence their prescribing decision, they welcome representatives to visit them and consider receiving free samples, gifts and various kinds of support as a normal practice. The findings provided insight into possible target areas for educational interventions concerning pharmaceutical marketing. Such a finding will provide the basis for policymakers in the public and private health sector in Yemen to develop a suitable policy and regulations in terms of drug promotion.

## Background

The pharmaceutical industry invests heavily in promotion, and it has used a variety of promotional strategies to stimulate sales of pharmaceutical drugs. Within this context, medical representatives (MRs) are the key personnel employed in promoting their products. According to reports, US$20.4 billion was spent on medical representative visits in the US in 2004 –35.5% of the total promotional spending [1]. An estimated 84% of pharmaceutical marketing is directed toward physicians. This includes items such as free samples, journal advertisements and visits from medical representatives [2].

The Yemen pharmaceutical market is an open market overseen by the Supreme Board of Drug and Medical Appliances (SBDMA). The number of registered medicines has reached 12,596. The pharmaceutical market was estimated to be worth US$297 million in 2010, compared to US$70 million in 2000 (a compound annual growth rate of 17.42%) [3]. One significant consequence of this rise in the number of pharmaceutical companies and available drugs is increased competition between drug companies. This increases the need for intensive marketing and promotional activities.

Except for the Essential Medicines List and the National Standard Treatment Guidelines for the most common illnesses which are narrowly distributed in primary care facilities, most of the prescribing takes place in private and public hospitals in addition to private clinics. However the physicians are not trained in evidence-based medicine, and neither a national formulary nor a drug information centre exists in Yemen.

Although the legal provisions prohibit direct-to-consumer advertisements in Yemen, but it’s still not yet implemented, there is no national code of conduct concerning drug promotion or guidelines for physicians’ interactions with MRs of pharmaceutical companies [4]. Previous studies found that most physicians report frequent meetings with medical representatives and a substantial percentage of physicians rely heavily on medical representatives as sources of information about new drugs [2,5,6]. Interactions between physicians and MRs are inevitable and desirable, but may create conflicts of interest for physicians [7]. One significant consequence of such relationships has been that they often result in a conflict of interest between a physician’s duties to their patient on the one hand and the pharmaceutical industry’s interest in maximising the sale of its products on the other, which may contribute to the over prescribing of medications and thus negative effects on patients’ health and the economy [1,8].

This interaction has been discussed in several studies. Some have focused on the ethical implications of these interactions and the potential for exploitation [2,5,9]; others have concentrated on policy [7] and the possible effects of restricting contact between medical representatives and physicians, and some studies examined the effects of education intervention on physicians’ attitudes and behaviours toward medical representatives [10,11]. Despite quantitative surveys of physicians, we have little detailed information concerning the reasons physicians interact with medical representatives; few qualitative studies explain how they rationalize these visits and the reasons for these interactions.

In all developed countries, few qualitative studies have investigated aspects of physicians’ interactions with medical representatives [12-15]. However, differences between developed and developing countries, especially bearing in mind Yemen’s status as an Islamic and Arab country with a different culture, language and health care system, raise the question of whether those findings would be relevant to Yemeni physicians.

In the context of Yemen, in addition to the absence of any comprehensive regulations controlling drug promotion and the lack of a clear mechanism to monitor the promotional activities of the pharmaceutical industry, there is very little information available regarding the dynamics of the relationship between MRs and physicians, and the physicians’ attitudes regarding such interactions.

Bearing the above concerns in mind, this study aims to describe the range of interactions between medical representatives and physicians, exploring physicians’ attitudes about interactions with MRs and their reasons for accepting the medical representatives’ visits. The findings will provide insight into possible target areas for educational interventions in pharmaceutical marketing. Such a finding will provide the basis for policymakers in the public and private health sector in Yemen to develop a suitable policy and regulations in terms of drug promotion.

## Methods

This study was part of a larger PhD study in which Yemeni physicians were surveyed about the influence of drug promotion techniques on their prescribing decisions. The focus of this study was on physicians’ interactions with MRs as one of the aspects of the present qualitative research. The current study was conducted with the range of physicians (intern, GPs/medical officers, residents and specialists) and was carried out in order to develop the questionnaire for the Yemeni context, to explore the ways in which findings might vary regionally and to compare the quantitative and qualitative results as part of a comprehensive study. An in-depth interview technique was used. An interview guide was developed based on a thorough literature review. The interview guide was tested in three pilot interviews with physicians who were not included in the final qualitative sampling frame. This study was conducted in Sana’a, the capital city of Yemen, between May and July 2009. Physicians from both private and public hospitals were selected using purposeful sampling, so as to cover the range of physicians (interns, GPs/medical officers, residents and specialists), size and location of practices and sex of physicians as far as possible. A total of 32 physicians from both private and public hospitals were interviewed. The physicians were visited at their respective offices to conduct the interview. The interviews focused on the following issues: the interaction between medical representatives and physicians, physicians’ attitudes toward these interactions and the MRs, and reasons for their accepting the medical representatives’ visits. Appropriate probing questions were used when necessary. In order to draw out more complete ideas from the participants, they were given the freedom to express additional views on the topic at the end of the interview sessions. The protocol of this study was approved by Yemen’s Ministry of Public Health and Population (MOHP) ethics committee. All interviews were audiotape recorded, with the permission of the interviewees. The transcribed interviews (transcriptions) were subsequently analysed using a “framework” method based on Schutz’s sociological phenomenological approach in order to capture the essence of practitioners’ actual reasons for accepting the medical representatives’ visits [16]. Schutz’s social phenomenology was used as it offers a descriptive and interpretive theory of social action that explores subjective experience. This theory was deemed suitable as it is in line with preserving the participant’s subjective point of view and acknowledging the context within which the phenomenon was studied. The interviews were analysed in order to discern significant meaning units in ideas, and aspects of physicians’ perceptions of MRs’ visits. These were grouped and then brought together in categories. Themes were identified from several thorough readings of all the transcripts by the researcher and verified independently by a person with cognate experience in qualitative research (a faculty member) [17,18]. Thematic content analysis with systematic and comprehensive coding was first employed to identify categories of physicians’ reasons for accepting the MRs’ visits. Framework analysis was employed as it offers a systematic approach [19]. Initially, in the familiarisation stage relevant items of text were highlighted while reading the transcript. This was followed by coding of the interview data through a provisional thematic framework identifying key issues, concepts and themes so that the data could be examined and referenced to themes. Thereafter, indexing was performed through applying the index of the thematic framework to the transcriptions. The researcher made comparisons between the interviewees’ responses, and data were rearranged in the charting process. During the mapping and interpretation stage, patterns and associations were found and explanations for the findings generated. Discrepancies were discussed with the supervisors before final categorization and conceptualization was agreed. The identified themes were verified by discussion among all the researchers. This study was conducted in Arabic and the results were translated into English. Subsequently, the translation of the results was confirmed by another translator who is familiar with both languages.

## Results

A total of 32 physicians were interviewed; among them were 4 females, more than half of them form public hospitals. Thematic content analysis of the interviews identified three overarching or core themes which were felt to capture the phenomenon of physicians’ perceptions of MRs’ visits as described in the raw data (Table 1). The results are presented in three sections below, based on a final coding framework, starting with physicians’ interaction with MRs, their reasons for receiving MRs’ visits and reasons for not receiving MRs’ visits.

**Table 1:** A final coding framework

**First-order theme**	**Clustered themes**	**Second-order theme**
Welcoming interactions with all MRs	Physicians’ interaction with MRs	Medical representatives’ visits
Selecting MRs to meet with		
Avoiding meetings		
Mutual benefits	Reasons for receiving MRs’ visits	
Perceived legitimacy of MRs as providers of information in general and convenient source to new information		
Moral duty; social contracts with MRs		
Commercial context	Reasons for not receiving MRs’ visits	
Obligations; lack of conviction about the product		
Lack of credibility of MRs		
Inappropriate timing of visits		
Scepticism toward MRs		

### Physicians’ interaction with MRs

The majority of the physicians we spoke to were routinely visited by MRs. The range of visits was (0–30)/week, with a median (IQR) of 5 (2–13). Most physicians accepted MRs’ visits regardless of their company origin or whether they planned to prescribe the representatives’ products. Physicians rarely avoid or refuse visits from MRs. Only one of the 32 physicians in the study has never received visits from MRs. In Yemen, there is no national regulation for drug promotion and no clear mechanism is available to monitor the drug promotional activities of the pharmaceutical industry.

In Yemen, there is no policy to accept the representatives or not. Do you understand? Therefore, first, it depends on my conviction in the product, communication with representative and extent of my satisfaction to him. Here in Yemen, the personal relationship is a very important indicator, but the medicine has to be excellent. (Dr 14)

#### Welcoming interactions with all MRs

Most physicians welcomed MRs’ visits and explained that they do as a normal practice. They did not recognize any conflict of interest as they thought that they were immune from being influenced by their interactions with MRs.

All of them are my friends, there is no day without meeting representatives. Every day I meet four or three representatives. (Dr 16)

We welcome any representative regardless [of] his company, even representatives of Danish companies that have been boycotted, because he is considered as our colleague, so I cannot refuse to meet him. We [have a] discussion and then we choose the suitable medicine for the patient. (Dr 30)

#### Selecting MRs to meet with

Although most physicians welcomed the visit of the MRs, some of them have their own criteria for choosing the MRs for regular visits such as personal style, company and the kind of drugs they offer etc.

I have specific criteria for selection. I mean, whether I like this representative or not, whether I am comfortable with him or not and whether his style is true or not true. Is he logical or not logical? There are companies that do not care about them. For example, a new company whose products are widely available such as popular products. I often do not meet them because they do not give us new ideas. (Dr 03)

I accept all of them. After the interview, I apologise to some of them and some of them, I am always in touch with. Representatives of “X Group” visit me regularly. They visit me more than two to three times a week. (Dr 22)

#### Avoiding meetings

Really, from the time that I came here to work, I [have tried] to avoid meeting them because my use is limited, but I have to meet my colleagues. I try to avoid the interview because I know that I will not prescribe his product. I am a surgeon and my use is limited. Just I have painkiller. There is no other choice. (Dr 26)

### Reasons for receiving MRs’ visits

#### Mutual benefits

Receiving beneficial patronage or financial support from MRs is considered a reason for physicians to accept MRs’ visits.

Sometimes we need representatives in providing some medicines that we need it, some books or bulletin. Really, they help us in getting books, CDs and lectures from abroad that provided by some companies. They support us on this side a lot. (Dr 03)

Business deals are done. Great business deals. Maybe the physicians meet representatives for business deals. This happens because of physicians’ financial situation. Representatives help [doctors] a lot and […] provide a lot of support, and his company is famous, so why [would I not] meet him? I mean when the efficiency of the medicine is equal. After that, the representative has an important role to convince the doctor to prescribe his product. For me, I do not reject anyone. (Dr 06)

#### Perceived legitimacy of MRs as providers of information in general and convenient source to new information

Physicians recognize the professional authority of MRs as information providers. For example, they deliver information about medicines’ indications, side effects and contraindications, and offer comparisons between one specific product and another. Some physicians cited scientific discussion about the qualities of drugs as reasons for receiving representative visits.

[It is positive] that they can inform us about new products […] being launched in the market for the first time. Secondly, we can [hear about] alternatives from other companies that have the same effectiveness, low cost and less side effects. (Dr 24)

The positive things: of course, for example, they give us some booklets and brochures to help us, do you get it. Because we do not have enough time to read. (Dr 27)

In the current situation, when representatives visit me, first, he explains the medicine because I do not have enough information about it. Shows the latest studies that have been conducted. He may show me information that I do not know at all. (Dr 21)

### Moral duty; social contracts with MRs

The physicians believed that friendship and social interaction play an important role in accepting MRs and they thought that the reception of the MR was their moral duty.

For me, I do not reject any one because, first, he is human. He knocks [on] my door, so I have to listen to him. He presents what he wants but I am not obliged to take what he has. Second, maybe he needs something else. (Dr 06)

I think friendship plays an important role. It means that if the representative is my friend, I have to meet him. I think the friendship is very important between physician and representative – more than the scientific relationship. (Dr 16)

Social relationships between physicians and MRs might influence physicians and led them to support MRs through prescribing their products in order to make sales; finally, this increases the MRs’ credibility with their employers.

The other side is to facilitate services for colleagues as they do this task [to support] their families. This refers to a social and economic situation for colleagues because he gets a payoff to spend on his family. (Dr 05)

### Reasons for not receiving MRs’ visits

Physicians cited many reasons for refusing to meet MRs, such bad experience with MRs and commercial context (e.g., disagreements about some commercial deal), obligations with other companies, lack of conviction about the product, lack of credibility of MRs and busy or inappropriate timing of visits. However, those reasons were mentioned under certain conditions and did not mean that physicians not accept MRs’ visits at all. Most physicians welcome MRs’ visit and some of them select MRs based on their own criteria, as mentioned above.

#### Commercial context

A minority of physicians reported that their reasons for refusing to meet MRs had a commercial basis, as physicians might encounter some conflict with certain companies due to disagreements about a commercial deal.

Representative is elusive person, he tries to convince physician to prescribe his product without giving any support. It is possible to stop prescribing his product forever. (Dr 22)

Sometimes there are some reasons. I hear that some physicians quarrel with some companies and they refuse to meet their representatives. (Dr 12)

The other thing, I may refuse to meet [a] representative if the owner of the company behaves with our colleagues [in an] inhuman or dishonourable [way], so this forces us to stop prescribing its product and prescribe a similar alternative that exists in the market. (Dr 05)

#### Obligations; lack of conviction about the product

Some physicians feel some obligation toward some representatives as a result of a previous deal or certain services. Consequently, they may avoid meeting other MRs.

If the representative meets me and I am not convinced in his product, he is trying to impose his product [on me]. Really, I avoid him as much as possible. (Dr 10)

Sometimes physicians accept representatives. Some physicians, for example, accept all representatives. I, for example, determine my relation with a specific company. I mean, for example, if [a] representative comes to me, I apologise and tell him that I prescribe a specific product. No need for new embarrassment. You have to determine your relation from the beginning. Then the representative will respect you. (Dr 08)

#### Lack of credibility of representatives

Unqualified MRs usually harm the profession as they cannot offer the necessary information about their product or the therapy area which the product was designed for.

Sometimes a representative does not have good information. He is not a representative. He is not experienced. Finally, I discover that he is not a specialist, just a promoter. He says, ‘I have a good product for blood pressure’. However, he does not know what blood pressure is… Sometimes he provides inadequate information due to lack of good knowledge. He acknowledges that his information is insufficient. If a physician ratifies [such a] representative, he will begin moving in the wrong direction. (Dr 03)

In some cases, the representative imposes on the physician to prescribe a certain product. We can prescribe it in rare cases for some diseases. Some representatives say, ‘I have certain amount of medicine in your pharmacy and it's not dispensing, prescribe, just one or two’. This forced us to refuse to meet him again, because he imposes [on] me to prescribe his product for any patient without any reason. (Dr 05)

#### Inappropriate timing of visits

Another issue created by representatives is the time that they select to visit the physicians. Many physicians, such as those who work in emergency departments or those who see a high number of patients, do not have enough time to meet, listen to or even interact with the representatives, and consider them as time wasters who distract them from doing their duty for their patients.

Their disadvantages are that sometimes my clinic is crowded. Inappropriate time particularly for example, they come to meet me in emergency unit and I am head of emergency. I am very busy and it is crowded, so because of time I cannot talk with them. In addition, they cannot explain everything in detail and I cannot understand their explanations. (Dr 16)

If I have patients, frankly I try to avoid meeting them, but if I am free, there is no objection to sitting together and talking. (Dr 01)

#### Scepticism toward MRs

Participants still doubt the role of representatives, with some accusing them of creating problems, harming the ethical reputation of the profession and, moreover, harming the patients’ welfare.

Being a colleague may affect me psychologically and may not be for the benefit of the patient. [He may give us an exaggerated description of the medicine]. He gives us a good impression of the medicine, but the company is not good and the efficacy of the medicine is not good. He gives us a good impression of the medicine, but it does not act as he describes it. (Dr 19)

Disadvantages of representative: they sometimes come to make an agreement. He says, ‘prescribe my product and we will support you financially’. It is dirty. This is crime. (Dr 21)

The story is based on mutual benefit. This is believed to be unethical; sometimes it affects the quality of the product and sometimes it is dangerous and I think [representative’s name] came to [use] bad promotion activities. Some representatives know their products properly, but he deals with medicines as a businessperson and encourages physicians to prescribe [a product] by tempting them financially. (Dr 03)

## Discussion

Most physicians routinely receive visits from MRs. They are considered reactive consumers. The MRs typically initiate the visit. Generally the physicians are willing to receive visits however their reasons for receiving these visits varied widely.

Despite that, almost all the literature shows that the information provided by MRs is often biased towards the promoted product [20,21]. In the Yemeni context, as is indeed the case in many developing countries, the MR is still the main source of information, especially regarding new drugs [22]. Most literatures agree MPs provided partial information on medicines, (similar finds from Sudan [23] and Libya [24]). However, physicians interviewed in this study had controversial views toward the quality of information provided, and this finding coincides with Prosser and Walley’s [12] findings. These findings may reveal the physicians need to be able to recognize and acknowledge the influence of pharmaceutical companies’ promotions in order to utilize the information provided by the industry appropriately to reach to scientific based prescribing decisions for the benefit of their patients. Governments should establish drug information centres, program for academic detailing to provide physicians with scientific information about drugs as well as establish role of clinical pharmacist in hospitals and faculties of pharmaceutical sciences.

Medical representatives are a key component of companies’ marketing strategies, and MRs’ success in securing visits to physicians depends as much on the marketing communication strategies employed by the MR and his or her ability to vary their style in accordance with physicians’ personalities [25]. The interactive communication is important and can be successful simply through offering high-quality information to establish credibility, establishing social and interpersonal skills and offering suitable gifts. Many of these were described as reasons that stimulate the physicians’ decision to accept MRs’ visits, and all can generate a sense of reciprocal obligation on the part of the recipient [13,26].

Some physicians have a negative attitude toward MRs and their marketing activities, perceiving them as attempting to influence their prescribing decisions and risking a negative impact on the patient, as mentioned in results section. Most physicians believe that they are impervious to marketing pressure. This study suggests that some interactions and cooperation between physicians and the MRs are regarded within a commercial context and this context is one of the explanations that physicians provided for accepting or refusing MRs’ visits, no difference in this between physicians in public and privet hospitals.

The increased level of competition between drug companies in their marketing activities has led to one significant consequence: the availability of different kinds of medicine in Yemeni pharmacies. Physicians cited this as one reason that made them refuse to see some MRs.

In the context of Yemen, social aspects and culture play a role in physicians’ interaction with MRs. Physicians stated repeatedly that social responsibility, or what is seen by physicians as social obligation, is usually the overriding reason why they accept MRs’ visits, and at the same time, is a barrier to refusing to see them. Furthermore, most physicians welcome interactions with all MRs; they cited that the main reason for refusing to see an MR would be the inappropriate time of a visit. Some of the physicians felt embarrassed about that. The findings had provided insight into possible target areas for educational interventions about the guidelines for physicians in interactions with industry.

Although some physicians have a negative attitude to MRs due to some MRs’ behaviour, and they described these behaviours as unethical, but welcome MRs’ visits and consider receiving free samples, gifts and various kinds of support to be normal and ethical, especially with regard to educational support such as the provision of books and magazines and support in attending medical conferences, etc. However, the influence of physicians’ interactions with MRs appeared multifactorial; social aspect, lack in access to drug information, low income of physicians, the absence of regulated pharmaceutical promotion, competition between drug companies and heavy promotion. Although the findings suggest physicians were aware that the MRs could influence their prescribing decision, they do not have knowledge of any code of ethics regulating interactions with the pharmaceutical industry, due to the lack of this regulation in Yemen [27]. This emphasize the need to establish laws, ethics and policies for drug promotion to enable monitoring and encouraging compliance by pharmaceutical companies, and their medical representatives, to a code of conduct and ethical marketing practices.

Although qualitative studies are always limited by their inability to be generalized, the lack of generalizability in the qualitative research technique in this study is not an issue seriously affecting the usefulness of the study as it is more concerned about the behavioural phenomena of each individual [28]. Therefore, findings of the study can be useful to shed light about the phenomenon all over the entire country of Yemen and those countries which have a similar health system. Another limitation of this study which should be considered is that, as in case of all self-reported data, it includes the risk of social desirability bias. Our study is limited in that it was largely exploratory and it focused on physicians’ attitudes about interactions with MRs and their reasons for accepting the medical representatives’ visits. Future research should explore ethical issues, like accepting financial promotion items from MRs. As well, the conflicts of interest surrounding this practice should be discussed from an Islamic perspective with physicians through in-depth face to face interviews in a separate study.

## Conclusions

Although physicians were aware that the MRs could influence their prescribing decision, they welcome MRs to visit them and consider receiving free samples, gifts and various kinds of support as a normal practice. The findings provided insight into possible target areas for educational interventions about pharmaceutical marketing. Such a finding will provide the basis for policymakers in the public and private health sector in Yemen to develop a suitable policy and regulations in terms of drug promotion. A national formulary will help physicians to prescribe approved medicine. SBDMA should encourage pharmaceutical companies to establish a national code of conduct and ethical marketing practices. Any attempt to establish a rule for restricting contacts between physicians and MRs should be preceded by education interventions as well as taking into account the reasons reported by physicians, as mentioned in the results. MOPHP should establish drug information centres and programmes for academic detailing to provide physicians with scientific information about drugs, as well as establish the role of the clinical pharmacist in hospitals and faculties of pharmaceutical sciences.

## Abbreviations

MRs: Medical representatives; SBDMA: Supreme board of drug and medical appliances; MOPHP: Ministry of public health and population.

## Competing interests

The authors declare that they have no competing interests.

## Authors’ contributions

MA conceived of the study design, generated the research question, conducted the interviews with respondents, audiotaped and transcribed the interviews, and was responsible for themes coding and content analysis, interpretation of the results and discussion and the drafting of the manuscript. AMH and MIMI made substantial contributions to the concept and design, the themes discussion, and were involved in reviewing and critiquing the manuscript for important intellectual content and contributed in finalizing the manuscript. All the authors gave their final approval of the version submitted for publication.

## Pre-publication history

The pre-publication history for this paper can be accessed here:

http://www.biomedcentral.com/1472-6963/13/331/prepub
